# Langerhans cell histiocytosis of the sternum

**DOI:** 10.1080/03009730802642360

**Published:** 2009-04-24

**Authors:** Hiroyuki Tsuchie, Kyoji Okada, Hiroyuki Nagasawa, Michihiro Yano, Hiroshi Nanjyo, Yoichi Shimada

**Affiliations:** ^1^Department of Orthopedic Surgery, Akita University School of MedicineAkitaJapan; ^2^Department of Pediatrics, Akita University School of MedicineAkitaJapan; ^3^Department of Pathology, Akita University School of MedicineAkitaJapan

**Keywords:** Bone, Langerhans cell histiocytosis, radiograph, sternum

## Abstract

We report a rare case of Langerhans cell histiocytosis involving the sternum. The patient was a 12-year-old girl presenting with anterior chest pain and swelling. Radiographs and computed tomography showed an osteolytic lesion in the sternum. Technetium bone scintigraphy revealed increased uptakes in the sternum, the greater trochanter of the right femur, and the right distal tibia. Incisional biopsy for the sternum lesion was performed, and the histopathologic diagnosis was Langerhans cell histiocytosis. She was treated with chemotherapy and the symptoms disappeared.

## Introduction

Langerhans cell histiocytosis (LCH) is a relatively uncommon disease suffered by infants and children. Many factors have been considered as the cause of this disease. Willman et al. described that LCH is probably a clonal neoplastic disorder with highly variable biologic behavior (1). Shannon et al. described that LCH is Langerhans cell accumulation and proliferation at a focal site by immunoregulatory dysfunctions because many patients of LCH show a decrease of suppressor T lymphocytes in the peripheral blood, thymic dysfunction, or hypergammaglobulinemia (2). However, no conclusion has yet been reached.

In 1987 the Writing Group of the Histiocytosis Society proposed that LCH has become the preferred term (3), replacing histiocytosis X which had been proposed by Lichtenstein in 1953 (4). This disease encompasses three disorders: eosinophilic granuloma (EG), Hand-Schuller-Christian syndrome, and Letterer-Siwe syndrome according to their clinical and pathologic features (4). Eosinophilic granuloma is the most common subtype, accounting for about 70% of LCH cases (5), but sternal lesions are quite rare (6–13). We report one patient with eosinophilic granuloma involving the sternum, with a literature review.

## Case report

**Figure 1.**
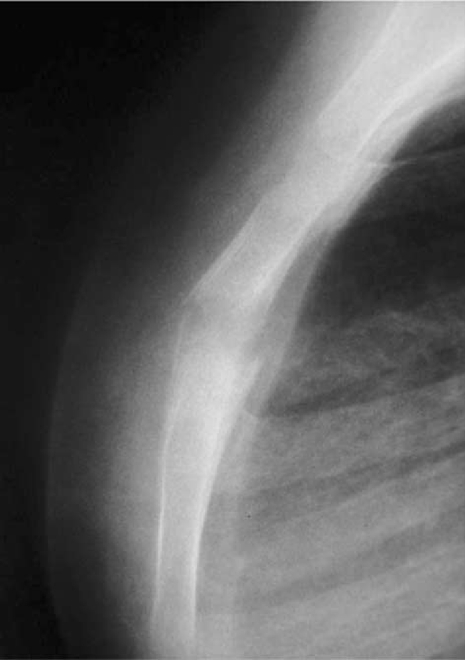
A lateral radiograph of the sternum showed an ill-defined osteolytic lesion in the sternum. Periosteal reactions around the lesion were also observed.

**Figure 2.**
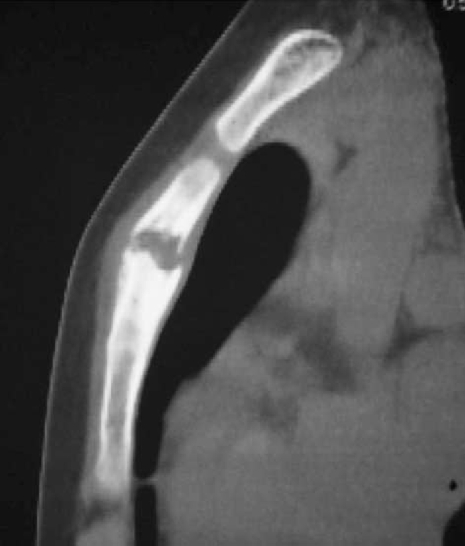
Computed tomography (CT) showed an osteolytic lesion that dissolved both anterior and posterior cortex of the body of the sternum.

**Figure 3.**
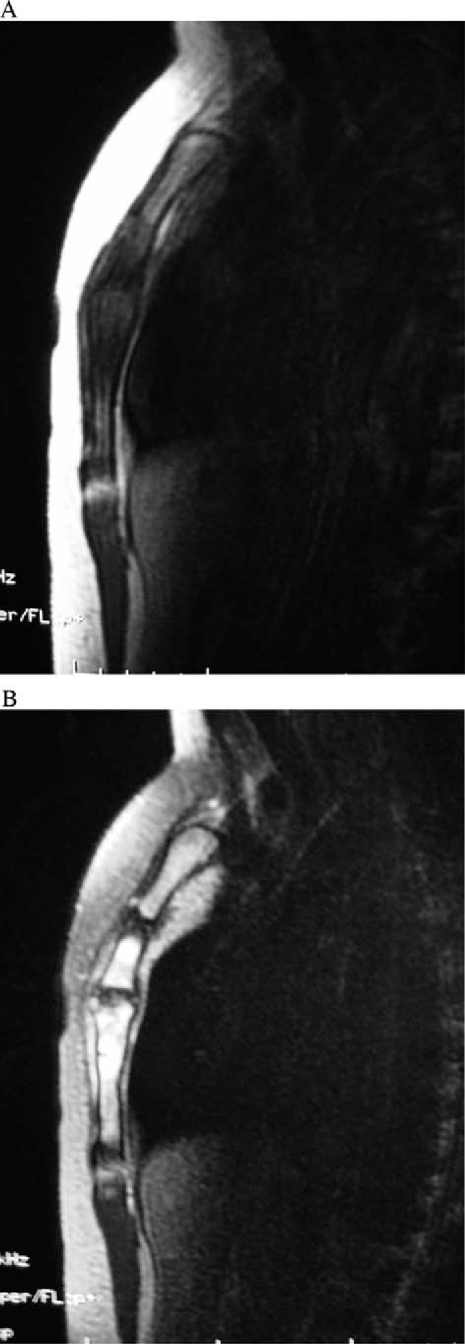
Magnetic resonance imaging (MRI). The signal intensity of the tumor was low in the sternum on T1-weighted images (A) and high on T2-weighted images (B).

**Figure 4.**
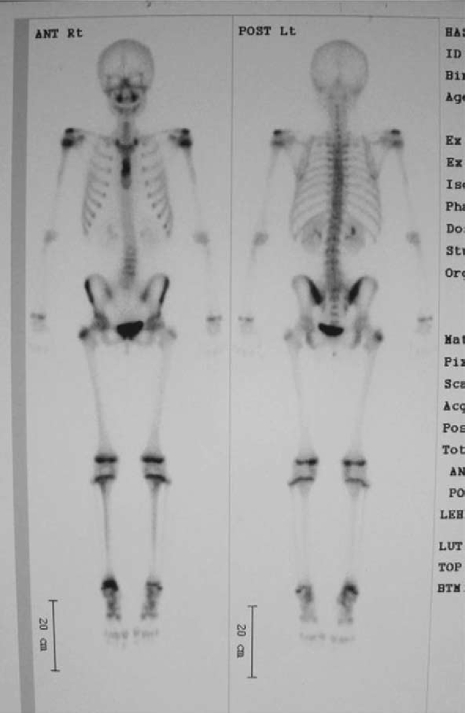
Technetium bone scintigraphy reveals increased uptakes in the sternum, the greater trochanter of the right femur, and the right distal tibia.

**Figure 5.**
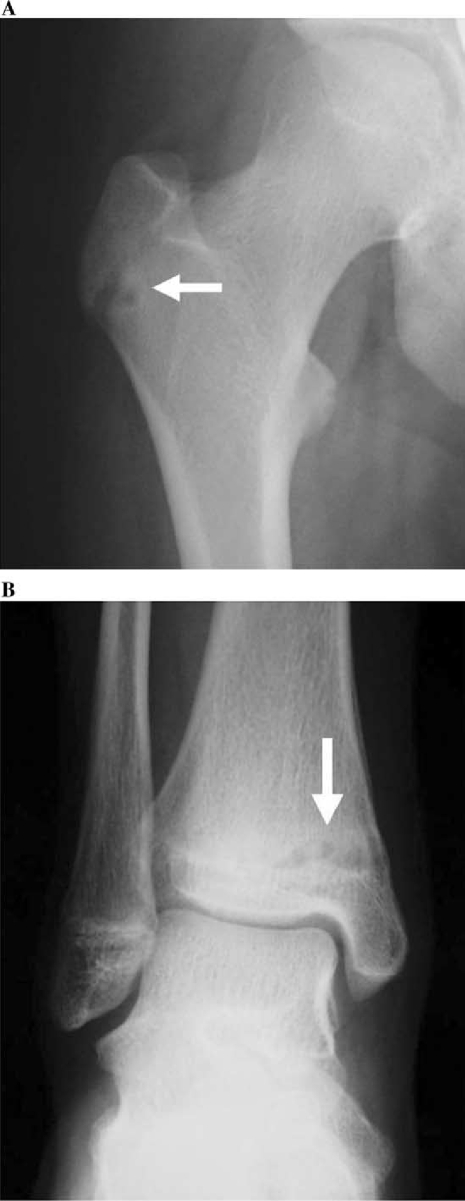
Radiographs show small and round osteolytic lesions (arrows) in the greater trochanter of the right femur (A) and the distal tibia (B).

A 12-year-old girl presented with anterior chest pain and swelling lasting 6 months. There was no history of trauma. This mass on the sternum was tender on physical examination. Radiographs showed an osteolytic lesion in the sternum (Figure 1). Computed tomography (CT) revealed the osteolytic lesion dissolving both anterior and posterior cortices of the body of the sternum (Figure 2). On magnetic resonance imaging (MRI), the lesion showed low signal intensity in the sternum on T1-weighted images and high signal intensity on T2-weighted images (Figure 3). Technetium bone scintigraphy revealed increased uptakes in the sternum, the greater trochanter of the right femur, and the right distal tibia (Figure 4). The greater trochanter of the right femur was tender, but the distal tibia was not, and radiographs showed small and round osteolytic lesions in both places (Figure 5). In blood test, the white blood cell count was normal (7,900 cells/mm^3^), but the rate of eosinophils was high (8.0%). C-reactive protein (CRP) of the plasma was normal (CRP: 0.37 mg/dL). There was no visceral lesion in the systemic survey.

**Figure 6.**
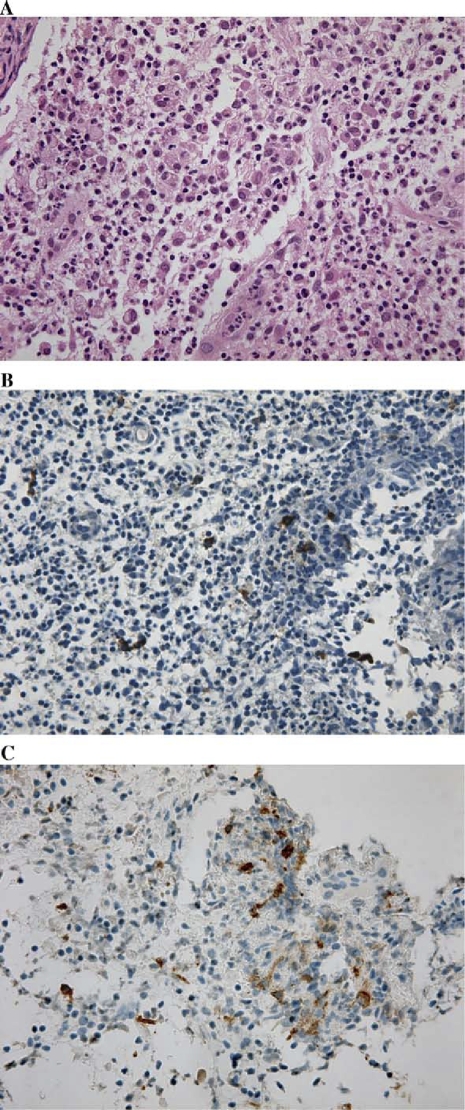
Histopathologic findings. Histopathologic examination reveals a proliferation of histiocytes with an infiltration of eosinophils. These histocytes were positive for S-100 (B), and for CD-1a (C). Original magnification ×400.

An incisional biopsy for the sternum lesion was performed in May 2008. Histopathologic examination revealed a proliferation of histiocytes with an infiltration of eosinophils. Immunohistochemically, these histiocytes were positive for S-100 and CD1a (Figure 6). A diagnosis of LCH was made.

Since the patient had the single-system multifocal type of eosinophilic granuloma, she was treated with chemotherapy without curettage of the bone (14,15). We used vincristine (0.05 mg/kg per day) and cytarabine (100 mg/m^2^ per day), according to the protocol of Japan LCH Study Group (JLSG), from May to June in 2008 (14). At the latest follow-up in July 2008, she had no complaint. MRI showed a decrease-of-signal change in the sternum lesion, and technetium bone scintigraphy showed decreased uptakes in the greater trochanter of the right femur and the right distal tibia.

## Discussion

Langerhans cell histiocytosis is an abnormal proliferation of tissue macrophages called Langerhans cells in one or more organs, including bone, skin, lymph nodes, lung, liver, spleen, and bone marrow. Patient age ranges from 5 to 15 years in about 90% of the cases, and males are slightly predominant (16). LCH represents less than 1% of tumor-like lesions of bone, and the majority are solitary lesions (79%) (17). The most frequent site is the skull, followed in decreasing order of frequency by the femur, jaw, pelvis, ribs, spine, scapula, humerus, and clavicle (18). Sternum lesions are extremely rare, representing less than 1% of bone lesions of LCH (18,19).

Only eight cases of sternal lesion of LCH have been reported in the literature (6–13). Of these eight cases, seven were well documented. Six of these seven were female. Age distribution was characteristic. Three of the eight were in the first decade of life, one was in the third decade, and the other three were in the fourth decade (Table I). The common complaints were pain, mass, and tenderness. Most cases of sternal lesion had a solitary lesion (6–13), and only one case had multiple bony lesions including the sternum (10). The multifocality of the bony lesions in the current case was characteristic.

**Table I.** Literature of sternal lesion of LCH.

Reference	Age	Gender	Lesions	Treatment
Wilson G J et al. (6)	6	F	Sternum only	Intralesional steroid
Sai S et al. (7)	25	F	Sternum only	Curettage
Fazio N et al. (8)	38	F	Sternum only	Partial sternectomy
Eroglu A at al. (9)	30	F	Sternum only	Partial sternectomy
Corti F et al. (10)	NS	NS	Sternum and other bones, skin	Radiotherapy and mecloretamin for skin lesion
Chiau JH et al. (11)	5	M	Sternum only	Curettage
Peer A et al. (12)	30	F	Sternum only	Radiotherapy and intralesional steroid
Gugliantini P et al. (13)	2	F	Sternum only	Chemotherapy

LCH = Langerhans cell histiocytosis; NS = not stated; F = female; M = male.

The common radiographic presentation for sternum in the reported cases was a purely osteolytic lesion without sclerotic margins. CT scanning defined the extent of the disease better than radiographs, confirming disruption of the cortex and extension of lytic lesion from the presternal to the retrosternal area (6,7,13). MRI showed hypointensive areas on T1-weighted images and hyperintensive areas on T2-weighted images. Similarly, in our case, radiographics showed the osteolytic lesion of the sternum, CT showed the extension of the lesion from presternal to retrosternal area, and MRI showed hypointensive on T1-weighted images and hyperintensive on T2-weighted images. The differential diagnosis includes osteomyelitis and several malignant bone diseases from aggressive radiological features such as cortical disruption and soft tissue extension. Although age, multifocality of the bony lesions, and increase of the rate of eosinophils indicated a diagnosis of LCH in the current case, we should stress that a diagnosis of LCH can only be finally made on histopathologic findings of a biopsy. Although we did not detect Birbeck's granules on electron microscopic study (20), an arrangement of histiocytosis in loose mesh-works or clusters (16) and immunoreactivity for S-100 and CD1a antigens are helpful for a diagnosis of LCH (21). When we encounter a sternal lesion, it might be difficult to remember the possibility of LCH because of its rarity. LCH should be considered in the differential diagnosis of patients under 15 who present with anterior chest pain and osteolytic lesion of the sternum.

Treatment of LCH depends on the extent of the disease. Various forms of treatment for a solitary lytic lesion affecting a long bone have been attempted. The therapeutic modalities include curettage, local steroid injection, radiotherapy, and chemotherapy alone or in combination, and the results of treatment of solitary lesions are always satisfactory finally (100% of isolated solitary bone lesions), although recurrence occurs in some patients (11% of them) (19). In contrast, multifocal and multisystem types of LCH are generally treated with chemotherapy, in combination with other therapeutic modalities. Since the current case was the single-system multifocal type of LCH, we performed chemotherapy with cytarabine and vincristine, and the effects were satisfactory.
